# Portal Hypertensive Polyposis in Advanced Liver Cirrhosis: The Unknown Entity?

**DOI:** 10.1155/2018/2182784

**Published:** 2018-08-01

**Authors:** David Kara, Anna Hüsing-Kabar, Hartmut Schmidt, Inga Grünewald, Gursimran Chandhok, Miriam Maschmeier, Iyad Kabar

**Affiliations:** ^1^Department of Gastroenterology and Hepatology, University Hospital Muenster, 48149 Muenster, Germany; ^2^University Hospital Muenster, Gerhard-Domagk-Institute of Pathology, 48149 Muenster, Germany; ^3^Department of Anatomy and Developmental Biology, and Neuroscience Program, Monash Biomedicine Discovery Institute, Monash University, Clayton, Melbourne, VIC 3800, Australia

## Abstract

**Background:**

Portal hypertension is a serious complication of liver cirrhosis.

**Objective:**

To identify relevant endoscopic findings in patients with advanced cirrhosis and consecutive portal hypertension.

**Methods:**

This was a retrospective study of liver transplant candidates who underwent upper gastrointestinal endoscopy between April 2011 and November 2015.

**Results:**

A total of 1,045 upper endoscopies were analyzed. Portal hypertensive gastric and duodenal polyps were frequently observed and were associated with thrombocytopenia (p = 0.040; OR: 2.4, 95% CI 1.04–5.50), Child-Pugh score > 6 (p = 0.033; OR: 2.3, 95% CI 1.07–4.92), Model for End Stage Liver Disease score > 16 (p = 0.030; OR: 4.1, 95% CI 1.14–15.00), and previous rubber band ligation (p < 0.001; OR = 5.2, 95% CI 2.5–10.7). These polyps often recurred after polypectomy; however, no malignant transformation occurred during the observational time until October 2017. The most common endoscopic finding was esophageal varices, observed in more than 90% of patients.

**Conclusion:**

Portal hypertensive polyposis is common in patients with advanced cirrhosis. Our data suggest that these polyps have benign characteristics.

## 1. Introduction

Portal hypertension is a common consequence and major complication of cirrhosis [1]. Portal hypertension is defined by an elevated portal pressure gradient caused by increased resistance to portal blood flow due to architectural changes in the cirrhotic liver, contraction of intrahepatic components as a result of decreased intrahepatic nitric oxide production, and increased splanchnic blood flow [1, 2]. Portal hypertension is a syndrome that involves several organ systems, leading to the formation of portosystemic collaterals, esophageal and gastric varices, gastropathy, enteropathy, colopathy, and splenomegaly with consecutive blood abnormalities including thrombocytopenia caused by hypersplenism [1].

In cirrhotic patients, endoscopy not only is used to detect esophageal varices but can also detect further gastrointestinal complications of portal hypertension such as portal hypertensive gastropathy or gastric varices. There have also been a few recent reports of polyposis related to portal hypertension [3–15]. The clinical relevance of this so-called portal hypertensive polyposis (PHP) remains unclear.

The present study was performed at a tertiary center and aimed to identify pathological findings during upper gastrointestinal endoscopy in patients with advanced cirrhosis who were under consideration for liver transplantation (LT) or who were already on the waiting list for LT in general and to explore the clinical characteristics of PHP in these patients.

## 2. Patients and Methods

This was an investigator-initiated, single center, retrospective analysis. All patients with cirrhosis who were under the care of the Department for Transplant Medicine at the University Hospital of Muenster and who underwent upper gastrointestinal endoscopy between April 2011 and November 2015 were considered for inclusion in this study. Inclusion criteria were the presence of liver cirrhosis, patient age 18 years or above, and available patient data. Patients' clinical and demographic data were collected from electronic healthcare files. All patients were regularly followed up at our outpatient clinic until October 2017. This study was approved by the Ethics Committee of the University Hospital of Muenster on April 28, 2016, and was carried out in accordance with the standards in the Declaration of Helsinki. Written informed consent was given by all patients prior to intervention.

### 2.1. Statistical Analysis

Statistical analysis was performed using IBM SPSS® Statistics 24 for Windows (IBM Corporation, Somers, NY, USA). Data are presented in both absolute and relative frequencies. Continuous variables with normal distribution are expressed as the mean ± standard deviation, whereas variables that do not follow normal distribution are shown as the median and maximal range.

Stepwise variable selection using univariable binary logistic regression analysis was performed to explore potential single risk factors for endoscopic findings. All variables that reached a significance level of p ≤ 0.1 were included in the multivariable binary logistic regression analysis to identify independent risk factors for the endoscopic finding being investigated.

## 3. Results

### 3.1. Study Population and Clinical Data

A total of 1,045 upper endoscopies performed in 407 cirrhotic patients were eligible for statistical analysis. The demographic data and clinical and laboratory characteristics of these patients are summarized in Table 1. Most of the patients were male. Mean patient age was 59 ± 11.2 years. The most common Child-Pugh category was B, followed by A and then C.

### 3.2. Endoscopic Findings

The endoscopic findings are summarized in Table 2. Esophageal varices were present in most patients. Grade I and II varices were present in 35.6% and 33.6% of cases, respectively, while grade III varices were found in 22.4% of patients. Gastric varices were found in approximately 10% of patients. Portal hypertensive gastropathy was as prevalent as esophageal varices and was observed in about 90% of patients.* Helicobacter pylori* was detected in 23 of the 219 patients (11%) in whom a biopsy was performed. Gastric polyps were present in about 10% of patients; these polyps mainly had the histologic characteristics of hyperplastic polyps, with foveolar hyperplasia and markedly proliferating, ectatic capillaries in the lamina propria. These portal hypertensive polyps were the most commonly found gastric polyps on biopsy and comprised more than 80% of all detected polyps. Adenomas were very rare (2.8%). Duodenal polyps were present in 8% of patients; these were also mostly hyperplastic. However, hyperplastic polyps were less frequent in the duodenum than in the stomach. Tubular adenoma and endocrine tumors were seen in two patients, with one patient showing a low-grade intraepithelial neoplasia.

An additional colonoscopy was performed in 363 of the 407 included patients. A total of 135 patients (37.2%) had evident colon polyps, of which 113 polyps were endoscopically removed. Of these 113 removed colon polyps, 71 (62.8%) were adenoma with low-grade intraepithelial neoplasia, 34 (30.1%) were hyperplastic, two (1.8%) were sessile adenoma, four (3.5%) were adenocarcinoma, and two (1.8%) were leiomyoma (Table 2).

### 3.3. Portal Hypertensive Gastric Polyposis

**Figure 1:**
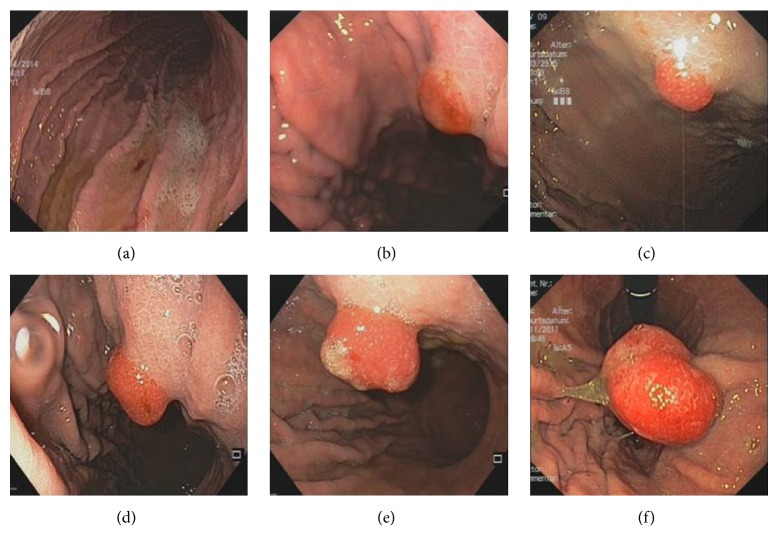
Chronological evolution of a portal hypertensive polyp in one patient after rubber band ligation of esophageal varices between 2014 and 2017 (a-f).

More than one polypoid lesion was present in the stomach in 79% of PHP cases. A total of 19 polypectomies were performed in 16 patients. There was no bleeding or perforation observed in any of the cases. Of the resected portal hypertensive polyps, 15 polyps (79%) recurred after polypectomy and were detectable in subsequent endoscopies. Notably, 55% of hyperplastic polyps with typical signs of PHP first arose or progressed following rubber band ligation (Figure 1).

**Figure 2:**
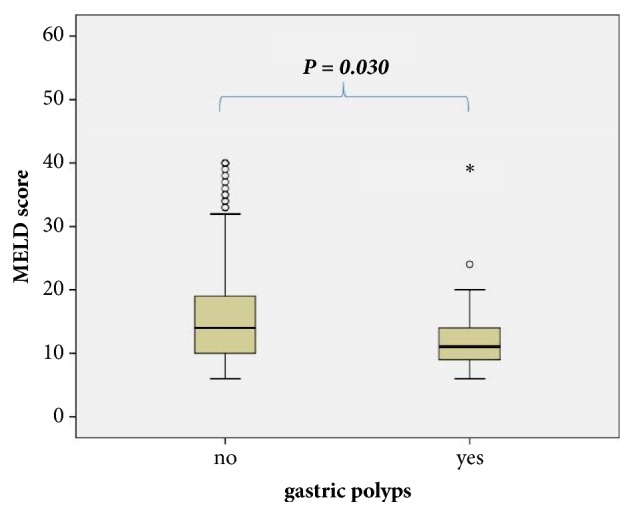
Distribution of gastric polyps with regard to MELD score. Higher MELD score was associated with the presence of hypertensive polyposis of the stomach, suggesting a higher prevalence of gastric polyps in advanced cirrhosis. MELD: Model for End Stage Liver Disease.

**Figure 3:**
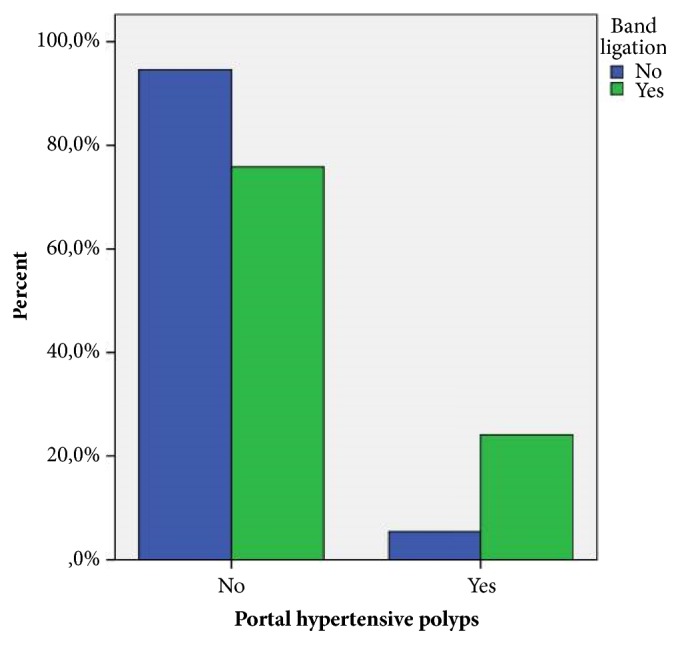
Bar chart showing the distribution of portal hypertensive polyposis with regard to rubber band ligation.

During a mean follow-up of 44.6 ± 14.7 months, none of the polyps degenerated into malignant carcinoma. No episodes of spontaneous bleeding related to portal hypertensive polyps were observed during the time of the study. All polyps were localized in the distal part of the stomach (antrum and prepyloric region). Using multivariable binary regression analysis, thrombocytopenia (defined as platelet count < 130 × 10^3^/*μ*l) was shown to be an independent risk factor of PHP (p = 0.040; OR = 2.4, 95% CI 1.04–5.50). The other independent predictors of the occurrence of PHP were Child-Pugh score > 6 (p = 0.033; OR = 2.3, 95% CI 1.07–4.92), MELD score > 16 (p = 0.030; OR = 4.1, 95% CI 1.14–15.00), and previous rubber band ligation (p < 0.001; OR = 5.2, 95% CI 2.5–10.7) (Figures 2 and 3).

In multivariable analysis, male sex (p = 0.01; OR 1.9, 95% CI 1.2-3.2), evidence of duodenal polyps (p = 0.02; OR 2.5, 95% CI 1.4-5.3), and HCC (p = 0.04; OR 1.8, 95% CI 1.0-3.1) were found to be significantly associated with colonic polyps.

Statistical analysis showed no association between proton-pump inhibitors and PHP (p = 0.680; OR = 0.946). However, it should be pointed out that the majority of patients received PPI. Therefore, the analysis regarding the role of PPIs may be limited by this fact.

Binary regression analysis showed no association between beta-blockers and PHP (p = 0.460; OR = 0.968)

## 4. Discussion

To the best of our knowledge, the present study is the largest study investigating upper gastrointestinal endoscopy findings in LT candidates with advanced cirrhosis. The present study identified a very high prevalence (> 90%) of esophageal varices and portal hypertensive gastropathy. Previous studies have estimated the prevalence of esophageal varices at the time of diagnosis of liver cirrhosis as about 35% in patients with compensated cirrhosis and 60% in patients with decompensated cirrhosis [1], while portal hypertensive gastropathy is reportedly seen in 11–80% of cirrhotic patients [1, 16–18]. Our patients underwent endoscopy at the time of referral to a tertiary center, which probably explains the greater frequencies of varices and portal hypertensive gastropathy. One study on LT candidates undergoing screening endoscopy reported incidences of varices and portal hypertensive gastropathy of 73% and 62%, respectively [17]. The higher prevalence of these findings in our study may be explained by the presence of more advanced disease in our cohort (Child-class C in 26% in our study versus 17% in the study by Zaman et al. [17]). The prevalence of both portal hypertensive gastropathy and variceal progression is strongly correlated with the increasing severity of cirrhosis [1, 17, 18]. The higher prevalence of these complications in our study may also partly be explained by the extensive experience of our endoscopists in endoscopic examination of patients with cirrhosis, as our center is highly specialized in this field, allowing the detection of early endoscopic alterations. The detection of grade I varices in nearly 40% of cases may be consistent with this assumption.

In our study, splenomegaly (83%) and ascites (56%) were also highly prevalent findings. This is consistent with another study on LT candidates [19].

### 4.1. Portal Hypertensive Polyposis

Apart from the expected cirrhosis-related pathologies such as esophagogastric varices and portal hypertensive gastropathy, there was a noticeable high prevalence of gastroduodenal polyposis observed in our patients. The prevalence of gastroduodenal polyps in the general population reportedly ranges from 0.5% to 6.35% [3, 4]. In contrast, gastroduodenal polyps were far more frequent in our study; almost 10% of patients had gastric polyps, and 8% had duodenal polyps.

**Figure 4:**
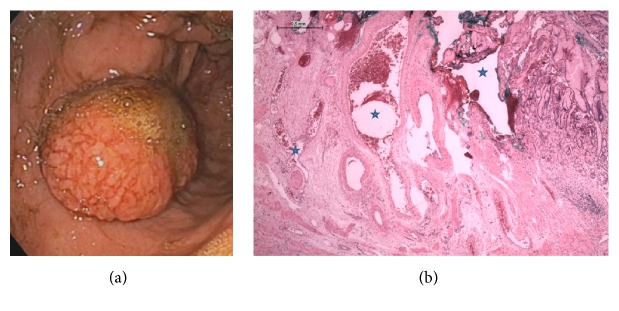
Images of hypertensive polyps. (a) Macroscopic aspect of a portal hypertensive antral polyp. (b) Histological image of a hypertensive gastric polyp showing proliferating ectatic vessels (starlets).

Gastric and duodenal hypertension has been associated with the presence of portal hypertensive polyps, but this has mostly been reported in case reports and a few small case series [5–10, 12–15]. In our study, we comprehensively evaluated the clinical appearance of PHP. These polyps are typically localized in the stomach; however, they can be found all through the intestine [8, 11, 14]. Macroscopically, portal hypertensive polyps cannot be distinguished from normal hyperplastic polyps but frequently present with small ulcerations [7, 9]. Even histologically, there are similarities between hyperplastic and portal hypertensive polyps [6]. There are still no clear diagnostic criteria for portal hypertensive polyps [11]. However, typical features of portal hypertensive polyps reportedly include foveolar hyperplasia of the epithelium as well as proliferating, ectatic capillaries in the lamina propria; this indicates their portal hypertensive nature and distinguishes them from inflammatory polyps (Figure 4) [3, 7, 8, 11, 14].

In our cohort, polyps were pathologically classified as “hyperplastic” in the majority of cases, even though they showed the abovementioned histological criteria of portal hypertensive polyps. One notable characteristic of these polyps in our study was that they almost always occurred in multiples. Other studies including cirrhotic patients have reported a PHP frequency of 0.9–1.3% [6, 8, 11]. As portal hypertensive polyps are still relatively unknown by both endoscopists and pathologists, they may be considerably underdiagnosed. The pathogenic mechanism of PHP remains unknown, but increased congestion caused by increased portal pressure may play an important role in inducing proliferation and angiogenesis. Some observations suggest that these polyps may respond to the treatment of portal hypertension [8–10]. Therefore, the presence of these portal hypertensive polyps may have been particularly high in the present study due to the advanced stage of cirrhosis in our cohort. Accordingly, the independent risk factors for PHP were identified as thrombocytopenia (platelet count < 130 × 10^3^/*μ*l), Child-Pugh score > 6, and MELD score > 16. Of note, the strongest risk factor for the development of these polyps was previous rubber band ligation. This may be because band ligation of the esophageal varices leads to increased formation of portosystemic shunts, including the gastric wall. This hypothesis is also consistent with the histological finding of proliferating ectatic vessels in the gastric mucosa and strongly supports our hypothesis of an evident proliferation stimulus of the increased portal blood flow on the gastric mucosa.

PHP is still poorly understood, and little is known about the risks and benefits of endoscopic resection. Although endoscopic resection was performed in all cases without complications in our study, the necessity of polypectomy should be critically considered, as portal hypertensive polyps frequently recurred and not one malignant transformation was observed during follow-up of 44.6 ± 14.7 months.

In our study, the incidence of colon polyps and the frequency of adenoma within these polyps were similar to those reported in another cohort of LT candidates (37% versus 42% for colon polyps and 54.1% versus 53.6% for adenoma within colon polyps) [20]. In contrast to the polyps found in the upper gastrointestinal tract, adenomas are the most common detected entity of colon polyps. As this entity represents a preliminary stage of adenocarcinoma, colon polyps should always be resected and studied histopathologically to assess their potential for malignant transformation. Subsequent endoscopic surveillance of colonic polyps depends on number, size, and histopathology of polyps, as well as the prevalence of hereditary conditions [21]. The Paris classification of gastrointestinal lesions can be used to classify colon lesions into polypoid, nonpolypoid, and depressed or excavated, where the latter is more likely to show high-grade dysplasia or malignancy (the Paris endoscopic classification of superficial neoplastic lesions: esophagus, stomach, and colon).

Some data indicate a positive association between higher levels of sex hormones in men and the development of colorectal carcinoma, while estradiol seems to have protective effects [22]. Given the adenoma-carcinoma-sequence, these findings may give an explanation for the higher rate of colon polyps in men in our patient cohort.

The higher prevalence of colon neoplasia in patients with the evidence of incidental duodenal polyps emphasizes the recommendation for colonoscopy in patients with sporadic duodenal neoplasia that has been stated in former studies [23, 24].

One interesting finding of our study was the positive association between HCC and the prevalence of colon polyps. This fact may be explained by the results of several studies indicating a higher rate of colorectal polyps in patients with liver cirrhosis, while liver cirrhosis is also the main risk factor of HCC [20, 25].

## 5. Conclusions

PHP is a common finding in patients with advanced liver cirrhosis, which until now may have been underestimated by both endoscopists and pathologists. These PHP lesions are typically localized in the antrum of the stomach, are mostly multiple, and show typical microscopic findings. Portal hypertension seems to play a crucial role in the pathogenesis of PHP, as these lesions are mostly seen in advanced cirrhosis, frequently after rubber ligation of preexistent esophageal varices. There is currently no evidence of these polyps having malignant potential. In our opinion and based on these findings, both polypectomy and endoscopic surveillance are dispensable in case of PHP.

## Figures and Tables

**Table 1:** Demographic data and clinical and laboratory characteristics.

	n = 407
Age [years], median (range)	60 (21–88)
Females/males, n (%)	127 (31.2%)/280 (68.8%)
Ethanol (active or past substantial consumption)	111 (27.3%)
Hepatitis C	77 (18.9%)
Non-alcoholic fatty liver disease	63 (15.4%)
Cryptogenic	40 (9.8%)
Hepatitis B	23 (5.7%)
Primary sclerosing cholangitis	21 (5.2%)
Autoimmune hepatitis	15 (3.7%)
Hemochromatosis	9 (2.2%)
Wilson's disease	7 (1.7%)
Primary biliary cirrhosis	5 (1.2%)
Miscellaneous	36 (8.8%)
Splenomegaly	328 (82.8%)
Ascites	228 (56.4%)
Encephalopathy	118 (29.0%)
I–II	103 (25.3% of all patients)
III–IV	15 (3.7% of all patients)
Thrombocyte count	101 (18–630) thousand/*µ*l
International normalized ratio	1.3 (0.86–4.80)
Creatinine	1 (0.10–16.60) mg/dL
Albumin	3.3 (0.20–4.80) g/dL
Bilirubin	3.9 (0.2–40.1) mg/dL
Child-Pugh score	
≤ 6	142 (34.9%)
> 6	265 (65.1%)
Child-Pugh class	
A	142 (34.9%)
B	158 (38.8%)
C	107 (26.3%)
Model for End Stage Liver Disease score, mean ± SD/ median (range)	15.2 ± 7.3/ 13 (6–40)
Portal vein thrombosis	35 (8.6%)
Hepatocellular carcinoma	78 (19.2%)
Beta-blocker	299 (73.5%)
Proton-pump inhibitor	378 (92.9%)

**Table 2:** Endoscopic findings.

**Gastroscopy**	n = 407
Esophageal varices	373 (91.6%)
Grade I	145 (38.9%)
Grade II	137 (36.7%)
Grade III	91 (24.4%)
Barrett‘s esophagus	28 (6,9%)
Gastric varices	40 (9.8%)
Portal hypertensive gastropathy	373 (91.6%)
Gastric polyps	38 (9.5%)
Histopathology of endoscopically obtained biopsies (n = 36)	
Hyperplastic	29 (80.6%)
Foveolar hyperplasia	3 (8.3%)
Tubular adenoma	1 (2.8%)
Inflammatory	3 (8.3%)
*Helicobacter pylori*	23 (10.5%) (n = 219)^*∗*^
Duodenal polyps	32 (7.9%)
Histopathology of endoscopically obtained biopsies (n = 22)	
Hyperplastic	10 (45.5%)
Tubular adenoma	2 (9.1%)
Inflammatory	1 (4.5%)
Brunner glands	4 (18.2%)
Lipoma	2 (9.1%)
Endocrine tumor	2 (9.1%)
LGIEN	1 (4.5%)
**Colonoscopy **	n = 363
Colon polyps	135 (37.2%)
Histopathology of endoscopically obtained biopsies (n = 113)	
Hyperplastic	34 (30.1%)
Hyperplastic and LGIEN	16 (14.2%)
LGIEN	55 (48.7%)
Sessile adenoma	2 (1.8%)
Adenocarcinoma	4 (3.5%)
Leiomyoma	2 (1.8%)

^*∗*^Only tested in 219 patients.

LGIEN = low grade intraepithelial neoplasia.

## Data Availability

The data used to support the findings of this study are included within the article.
